# Peribronchial Consolidation with Surrounding Ground-Glass Opacity in COVID-19 Pneumonia: 3D Reconstruction of a Chest Computed Tomography

**DOI:** 10.4269/ajtmh.20-0405

**Published:** 2020-05-14

**Authors:** Michaela Cellina, Marcello A. Orsi, Giancarlo Oliva

**Affiliations:** Department of Radiology, ASST Fatebenefratelli Sacco, Milan, Italy

**Figure 1.**
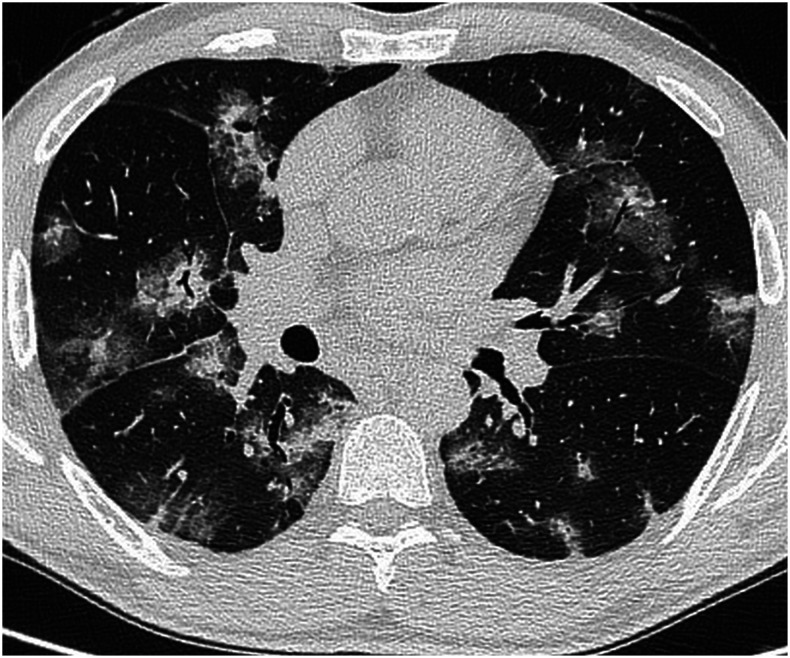
Chest computed tomography showing bilateral nodules and consolidations, surrounded by ground-glass opacities, and resulting in a halo sign, with prevalent peribronchovascular distribution. Air bronchograms are bilaterally recognizable.

**Figure 2.**
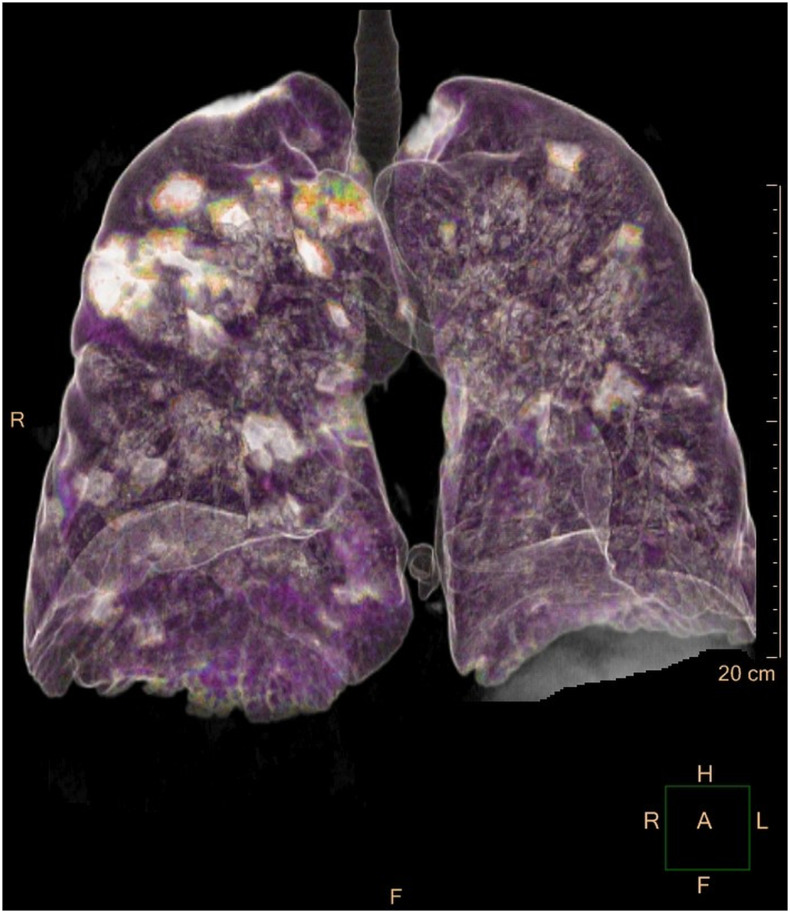
Volume-rendered three-dimensional reconstruction chest CT image shows the presence of bilateral opacities, with prevalent peribronchovascular distribution and sparing of the peripheral lung zones.

A 54-year-old man presented with a 5-day fever, cough, dyspnea, and chest pain. Physical examination revealed bilateral crackles on lung auscultation; temperature was 39°C. C-reactive protein was 27.2 mg/L (normal range: 0–5 mg/L) and serum lactate level was 274 U/L (normal range: 135–225 U/L); other blood tests showed normal results. COVID-19 was detected in two oropharyngeal swab samples by RT-PCR on consecutive days; *Legionella* and *Streptococcus pneumoniae* urinary antigens and PCR for other respiratory viruses on nasopharyngeal swabs were all negative. A bronchoalveolar lavage excluded pulmonary aspergillosis. On day 3, the patient developed severe dyspnea with decreased oxygen saturation (90%). Unenhanced chest computed tomography (CT) imaging showed diffuse bilateral peribronchial consolidations surrounded by ground-glass opacities (Figures 1 and 2).

The typical CT pattern of COVID-19 pneumonia consists of ground-glass opacities (GGO) with bilateral and peripheral distribution.^1^ However, less common imaging findings have also been reported. A pattern of peribronchial infiltrate with surrounding GGO is not well described in COVID-19, let alone to this extent.^2^ Radiologists should be aware of the wide spectrum of CT manifestation of this infection.
